# Distribution and morphological variation of tree ferns (Cyatheaceae) along an elevation gradient

**DOI:** 10.1371/journal.pone.0291945

**Published:** 2023-09-27

**Authors:** Gabriel Merino, Santiago Ramírez-Barahona, Mark E. Olson, Juan Núñez-Farfán, Felipe García-Oliva, Luis E. Eguiarte

**Affiliations:** 1 Departamento de Ecología Evolutiva, Instituto de Ecología, Universidad Nacional Autónoma de México, Mexico City, Mexico; 2 Posgrado en Ciencias Biológicas, Universidad Nacional Autónoma de México, Unidad de Posgrado, Ciudad Universitaria, Coyoacán, Mexico City, Mexico; 3 Departamento de Botánica, Instituto de Biología, Universidad Nacional Autónoma de México, Mexico City, Mexico; 4 Instituto de Investigaciones en Ecosistemas y Sustentabilidad, Universidad Nacional Autónoma de México (UNAM), Morelia, Michoacán, Mexico; KGUT: Graduate University of Advanced Technology, ISLAMIC REPUBLIC OF IRAN

## Abstract

Knowing how species and communities respond to environmental change is fundamental in the context of climate change. The search for patterns of abundance and phenotypic variation along altitudinal gradients can provide evidence on adaptive limits. We evaluated the species abundance and the variation in morphometric and stomatal characters in five tree ferns species (*Cyathea fulva*, *C*. *divergens*, *C*. *myosuroides*, *Alsophila firma* and *Gymnosphaera salvinii*) distributed along an elevation gradient in a well-preserved Mexican cloud forest. Variation at the community and species level was assessed using exploratory and multivariate data analysis methods. We wanted to explore if the species abundance is environmentally determined, to determine the degree of variation along the elevation gradient, to test for differences between zones and associations with elevation, humidity and soil nutrients, and to assess contribution of the intra- and interspecific variation to the community response to elevation and soil nutrients. The studied fern community showed strong species turnover along the elevation gradient, with some influence of soil nutrient concentration, supporting environmental determinism. All measured characters displayed variation along the gradient. Stomatal characters (size and density) had significantly less variation than morphometric characters (trunk diameter, stipe length and blade length), but stomatal density also shows interesting intraspecific patterns. In general, patterns within the fern community suggest a strong influence of species identity, especially of species inhabiting the lower edge of the cloud forest, which showed the clearest morphometric and stomatal patterns, associated to contrasting environments rather than to changes in elevation. The coincidence between morphometric and stomatal patterns in this area suggest hydraulic adjustments in response to contrasting environments. Our results provide evidence that tree ferns species respond to environmental changes through adjustments of morphometric plasticity and stomatal density, which is relevant to predict possible responses to variation in environmental conditions resulting from climate change.

## Introduction

Knowing how populations, species and communities respond to climate change is currently of fundamental interest in biology to develop better conservation strategies. Natural environmental gradients are an ideal setting to assess such a response through the search for patterns of species richness, species turnover, and genetic/phenotypic variation [1–4]. The strategies displayed by organisms to adapt to environmental change (e.g., temperature, humidity, pH, salinity, and soil nutrients) are limited [2,3,5–9] and affect species distributions [2,10,11] partly as result of environmental determinism and niche conservatism [12,13].

Genetic and phenotypic variation along environmental gradients generally shows patterns that are specific to different levels of organization along the biological hierarchy (e.g., species, communities). Factors that determine patterns of variation at the species level may not have an effect on attributes at the community level, which may otherwise be affected by other factors such as interspecific interactions [14]. Likewise, focusing on higher levels of organization (e.g., inter-specific variation) can miss the role of intra-specific variation on community structure and its responses to environmental change [15,16]. Therefore, establishing links across the levels of the biological hierarchy is desirable to develop integrative studies of biological variation and its drivers [14].

In mountainous regions, elevation gradients showing rapid environmental change along small geographic areas have been useful to assess patterns of morphological variation within plant communities [17–20]. Evidence on variation in leaf morphological and physiological traits suggest that the contribution of inter- and intra-specific variability to plant community responses to environmental variation differs across gradients (e. g., soil properties, restoration, elevation), ecosystems, and studies [15,16,21–23]. Furthermore, this evidence does not include studies on tropical forest in the New World. In the case of tropical montane forests (e. g., cloud forest), patterns related to elevation may overlap with those related to relative humidity or soil nutrients [24,25].

Among species that inhabit montane regions in the Neotropics, tree ferns (Cyatheaceae) conform a distinctive and conspicuous community, especially thriving within highly threatened cloud forests. Tree fern communities are of great importance in the structure and composition of the forest [26], in which they are frequently a dominant component, with a distribution along wide ranges of elevation within which they contribute an extraordinary diversity and a considerable level of endemism [27]. In addition, tree ferns are ecologically important because their role in nutrient cycling, control of ground-level irradiance and early succession as pioneer species capable of occupying forest gaps or edges [28,29], especially during forest regeneration events as a result of canopy disturbance phenomena [29,30], also facilitating epiphytic regeneration of tree species that inhabit their trunks [30,31].

Tree ferns often show strong patterns of altitudinal turnover among closely related species and varieties/morphotypes within species [32–34]. Different studies have evaluated the phenotypic variation of tree fern species associated with environmental change, including variation in stomatal characters [35,36], leaf traits [28,37–39], and plant size [37,40,41]. In addition, the study of phenotypic variation along elevation gradients has been useful to examine trait-environment relationship across both species and lineages [42]. This information is crucial to understand how environmental selection drives phenotypic adjustments in traits that impact on the fitness of individuals, promoting an efficient exploitation of resources in a given environment, and how consistent are those adjustments across lineages [43]. At the same time, it allows us to understand how the differential use of resources supported by the trait-environment relationship allows species to coexist in an area, impacting the patterns of diversity and differentiation observed along strong environmental gradients, thus defining the composition and structure of communities [44]. Despite this, few attempts have been made to assess trait variability at the community and species level along elevation gradients [18,42], showing that morphological variation tends to be more pronounced at the community level, except for stomatal traits, which varied more strongly at the species level.

In this study, we assess intra- and inter-specific variation in two stomatal (density and size) and three morphometric traits (trunk diameter, blade length and stipe length) in five species of tree ferns (*Cyathea fulva*, *C*. *divergens*, *C*. *myosuroides*, *Alsophila firma* and *Gymnosphaera salvinii)* distributed along a 1200 meter elevation gradient in the montane tropical forests of the Sierra of Juarez (Oaxaca), Mexico. Our main aim was to identify intra- and inter-specific patterns of variation associated with the observed patterns of species turnover at the community level considering elevation as proxy for temperature and soil nutrients as predictor variables, as well as relative humidity. Our specific aims were to: (1) explore whether species abundance is related to elevation and soil nutrients; (2) describe the degree of variation in stomatal and morphometric traits at the species and community levels, and assess their relationships with environmental gradients; (3) assess the contribution of the intra- and inter-specific variability of each character to the community response to elevation and soil nutrients.

## Materials and methods

### Sampling sites

**Fig 1:**
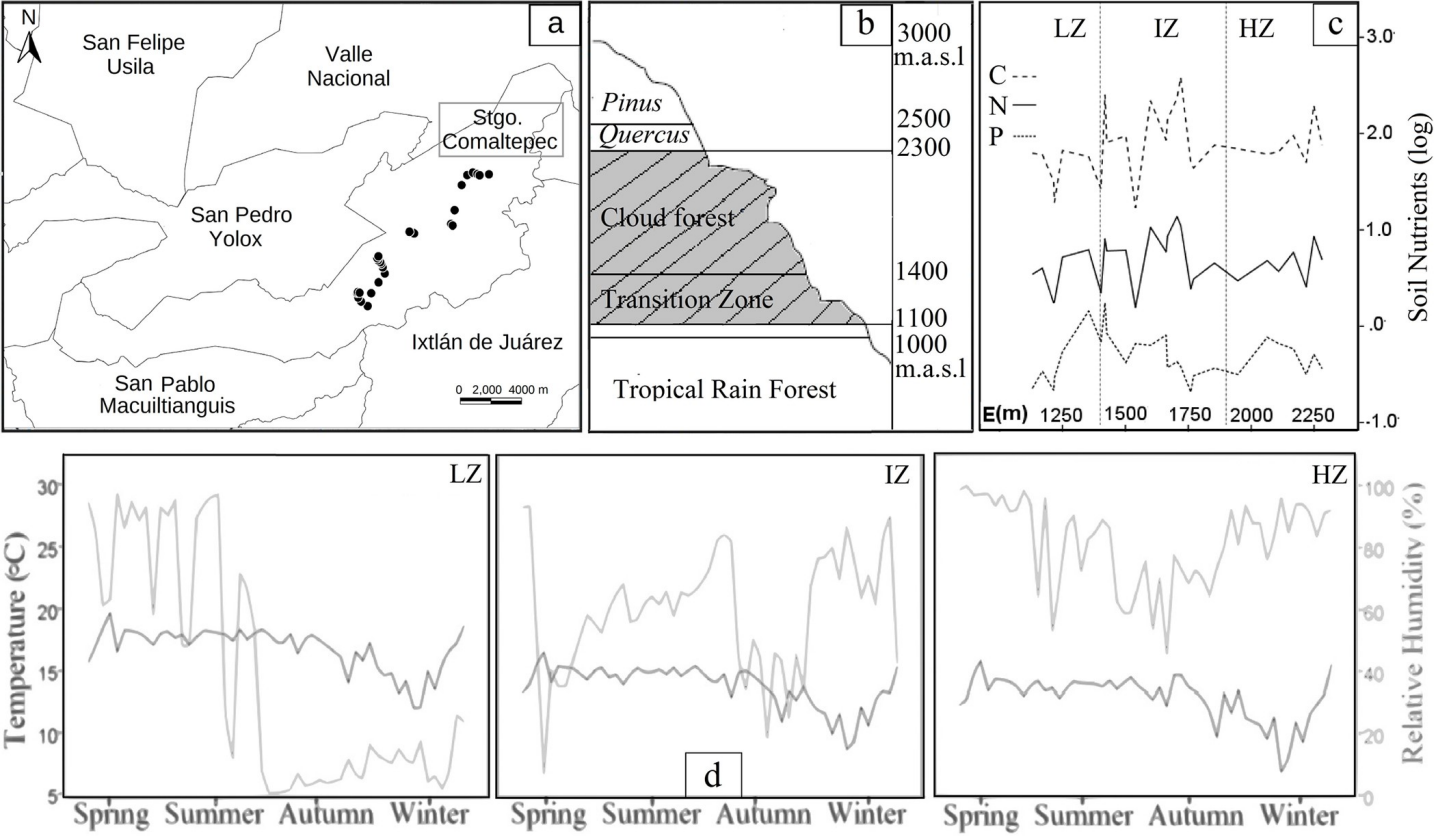
Elevation gradient localized at Sierra of Juarez, Oaxaca, Mexico. **a**. Geographic localization in the municipality of Santiago Comaltepec. The map was created using the software QGIS (version 3.12.2-Bucuresti). **b**. Vegetation profile of the elevation range. **c.** Concentration of three main soil nutrients along the gradient. **d.** Weekly measurements of relative humidity and temperature for three elevations reflecting environmental conditions of three zones (low, intermedial, high).

The sampling sites were located in the municipality of Santiago Comaltepec, Oaxaca, in a steep hillside that harbors the greatest number of tree fern species in Mexico (8–10 species) along an elevation gradient spanning 1,100–2,300 masl (Fig 1A). We established 26 sites along the elevation gradient with a 50-m difference in elevation between successive sites. Five main trails (“Relámpago”, “San Bernardo”, “La Esperanza”, “Vista Hermosa” and “Puerto Antonio”) were covered to achieve the desired elevation intervals, but for inaccessible elevation intervals we established roadside sites. Due to the steep slope, it was extremely difficult to establish replicate sites along the gradient. This made it impossible to test the consistency of species turnover pattern at different locations. Despite this, the sampling scheme resulted in the inclusion of pairs of species that shared the same elevation range (or significant intervals). This allowed us to evaluate the consistency of both the morphological patterns along the elevational gradient at the species level and the pattern of species turnover, the latter by identifying factors that limit species distribution at the inter-specific level. The areas above 2,300 m are dominated by Q*uercus* and *Pinus* and were not included in this study due to the absence of tree ferns; the area below 1,100 m (tropical rain forest) was not included due to the high human disturbance of the landscape [45].

We placed nine data loggers (HOBO U23 Pro v2) at three sites along the elevation gradient (1,400, 1,900, and 2,300 meters above sea level (masl hereafter); three loggers per site) to register hourly temperature (°C) and relative humidity over one year (2015–2016); recorded values were downloaded using the program HOBOware v3.7.14 (Onset Computer Corporation 2002–2018). Since temperature is linearly related to elevation [46], an interpolation of temperature was performed for the other sites along the gradient. The elevation gradient can be divided into two zones according to vegetation type: highly conserved cloud forests (CF: 1,400–2,300m) and a transition zone between tropical rain forest and cloud forest (TZ: 1,100–1,400m) (Fig 1B). Based on the climatic data, the elevation gradient was divided in a high zone (HZ), an intermediate zone (IZ), and a low zone (LZ), reflecting differences in temperature (°C) and relative humidity along the gradient (Fig 1D). The temperature presented variation in all the zones along the year, decreasing towards autumn and reaching lowest values in the winter. Mean temperature at the top of the HZ (2300 m), IZ (1900 m), and LZ (1400 m) was 13.0, 15.2, and 17.1°C respectively. Data loggers were lost for the plot at 1100 m, but interpolation suggest an approximate measure of 18.9°C. At 1400 m (the top of the transition zone), relative humidity dropped dramatically from summer to winter. The RH data obtained here appear to be severely affected by a relatively unexpected weather event. At the Sierra of Juárez, the forest structure is negatively affected by strong winds (> 80 km / h) about once every 10 years [47]. These strong winds cause massive tree falls and landslides, changing environmental and landscape conditions in the different zones of the elevation range (S1 Fig). In our study, such climatic event could explain the unexpected low RH values obtained by the data loggers, which makes characterization of the gradient difficult. However, based on our accumulated experiences from previous visits, we believe that differences shown here reflect, at least, clear differences in RH between the cloud forest (CF; 1400–2300 m) and the transition zone (TZ; 1100–1400 m).

Soil nutrient concentrations per site were characterized using six variables: total carbon (C), total nitrogen (N), total phosphorus (P), and C:N:P ratios; soil samples were taken by triplicate per site. Each sampling point was located 30 cm away from any tree fern and soil samples were taken at 30 cm of depth; soil samples were stored at -60° C. Soil nutrient concentrations were analyzed at the Instituto de Investigaciones en Ecosistemas y Sustentabilidad de la Universidad Nacional Autónoma de México (Morelia, Michoacán). Soil nutrients did not show significant differences between zones or vegetation types (S1 Table). Soil C and N showed a very similar behavior along the gradient (r = 0.931, p < 0.001) with the highest mean values in the IZ (1400–1700 m), in the lowest part of the cloud forest; the highest average soil P values were found at the top of the LZ (Fig 1C), which marks the transition zone into cloud forest. Soil P showed a weakly, but significant correlation with C (r = 0.198, p < 0.001) and N (r = 0.350, p < 0.001). Shared patterns of soil C and N, as well as differences with soil P, are well documented in the literature with consistency at different spatial scales [48–50]. These patterns are supported by a similar origin of C and N, both strongly determined by the organic matter in the soil, with external inputs occurring in ecological timescales, while P has an origin in bedrock and the weathering of primary minerals [51], with negligible external inputs occurring at that scale [50]. The variation in C:N:P ratios along the gradient is illustrated in S2 Fig.

### Tree fern sampling

Five tree fern species belonging to three different genera were sampled: *Cyathea fulva*, *C*. *divergens*, *C*. *myosuroides*, *Gymnosphaera salvinii* and *Alsophila firma*. Other tree fern species (*C*. *schiedeana* and *Sphaeropteris horrida*) were registered in the study area, but not sampled due to their low abundances (< 5 individuals). At each sampling site, we measured and sampled all tree fern specimens within a 10 x 15 m plot. Three morphometric traits were measured in the field: blade length (BL), stipe length (SL), and trunk diameter at breast height (TD); BL and SL were measured from one mature frond per plant and TD was estimated as perimeter / π.

Three pinnae were sampled from a mature frond per plant (at the base, center, and tip) and then dried and stored at room temperature until analyses. Two pinnules were taken from each pinnae and processed for microscopic analysis by applying nail polish to the abaxial side and removed when dried. These impressions were observed under the microscope to measure stomatal density (SD) and size (SS). Digital images were captured and analyzed with ZEN software v.1.0 (Zen 2011, blue edition, Carl Zeiss MicroImaging GmbH, 2006–2011). Three microscopic fields per pinnule were observed at 20x magnification and stomatal counts were performed and registered as number of stomata per mm^2^. Stomatal density was estimated as the product of cell guard length and width of closed stomata (μm^2^) for six stomata per individual.

### Statistical analysis

Species abundance data were recorded to characterize community composition along the elevation gradient and for the subsequent multivariate analysis. To assess the correspondence between abundance and environmental conditions, a canonical correspondence analysis (CCA) was performed. Both a species abundance matrix and an environmental measures matrix (elevation and soil nutrients) were used. The analysis was performed with the CCA package [52] in R (v4.0.5) [53].

Morphometric and stomatal traits were averaged across species and sites. A Shapiro-Wilk test was applied to verify normal distribution of data. To perform statistical analyses, the data were log transformed. Partial correlation analyzes were performed to test the association among traits. Analyzes were conducted controlling for all other traits to avoid effect of covariance. In addition, to find out whether the differences between species influence the correlations between traits, another set of paired correlations was obtained controlling for the species identity. A paired-samples *t*-test (two tailed) was performed to assess the differences between both sets of correlation coefficients.

Differences in traits along the gradient were evaluated using linear mixed-effects models (LMM). Three separated analyzes were carried out per character to assess differences between species, zones, and vegetation types. Each morphometric and stomatal trait was defined as the response variable, whereas zone and vegetation type were defined fixed factors, and species identity and site were defined as random factors. For each response variable, correlated morphometric and stomatal variables were used as covariates. The Games-Howell method was used as *post hoc* test among species. Additional linear mixed-effects models (LMM) were fitted for each species independently. All regression analyses were performed using the *lme4* package [54] in R (v4.0.5) [53]. Simple linear models (elevation as fixed factor) were compared to models containing random factors with the Akaike Information Criterion (AIC) using the MuMln package [55].

A principal component analysis (PCA) was implemented to examine the extent of variation of each trait with respect to the total variation along the elevation gradient and to obtain information on the interdependence of the traits examined. The PCA was done using PAST v. 3 [56]. A partial least squares regression (PLSR) was performed to assess the association between traits and soil variables. Morphometric and stomatal traits were defined as response variables, whereas elevation, C, N, P, and C: N: P ratios were defined as predictor variables. This analysis was carried out using the *pls* package [57] in R. Additional PLSR analyses were performed for each species.

### Contribution to community response (sum of squares analysis)

The contribution of intra- and interspecific variability to the community response to environmental gradients was evaluated for each morphometric and stomatal trait following Lepš et al. [15]. Abundance-weighted and unweighted averages of trait values were considered following Kichenin et al. [16]. This method decomposes the total variance at the site level (sum of squares) of any variable associated with an environmental variable (here, elevation and soil nutrients) into three components: Specific sum of squares = fixed sum of squares + intraspecific sum of squares + covariation sum of squares (Equation 1.1). Specific trait values were calculated per site by averaging species traits within each site, reflecting the combined contribution of inter-specific (species turnover) and intra-specific effects. Fixed traits values per site were estimated by averaging species traits across all plots in which the species occurs, reflecting the contribution of species turnover (inter-specific effects). Intra-specific trait values were estimated by subtracting the fixed trait values from the specific trait values. Regression analyzes were performed for each trait to estimate the sum of squares. Finally, the covariance between intra-and inter-specific variability was calculated using equation 1.1.

## Results

### Species abundances

**Fig 2:**
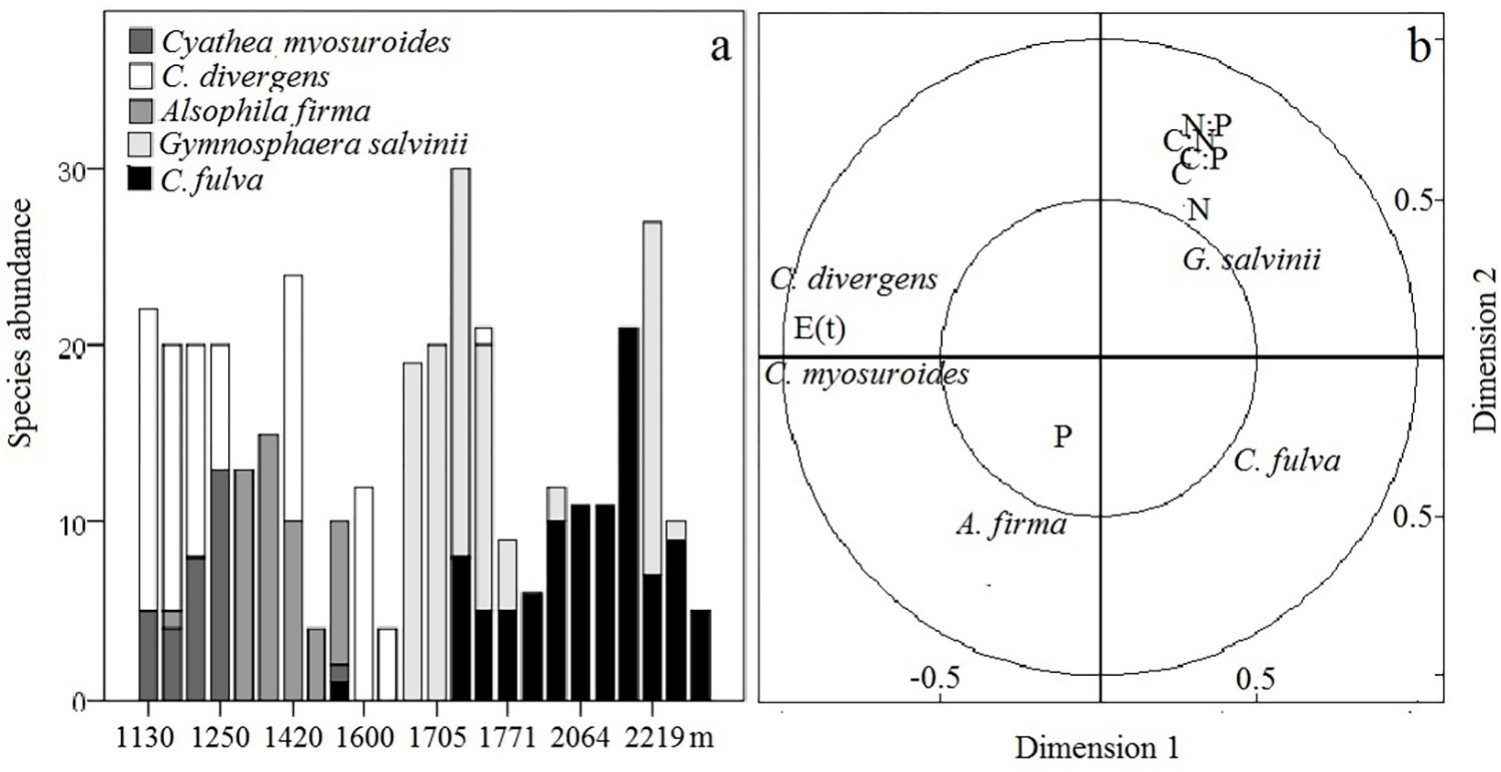
Abundance analysis for five tree ferns species along an elevation gradient in Sierra of Juarez (Mexico). a) Species abundance per plot. b) Canonical correspondence analysis showing the association between species abundance and environment (elevation and soil nutrients). Elevation was considered as a proxy of temperature. E. Elevation. t. Temperature. P. Phosphorus. C. Carbon. N. Nitrogen.

Tree ferns showed strong species turnover along the elevation gradient (Fig 2A). Some species shared specific zones along the gradient, but even with this overlap, a large percentage of plots (54%) included only one species. In the lower zone, *Cyathea myosuroides* and *C*. *divergens* co-inhabit all plots, whereas *Cyathea divergens* and *C*. *fulva*, the phylogenetically closest species, did not share any plots; the former was found at ~1700–2300 m and the latter below 1700 m. The CCA analysis showed that the abundance of *Cyathea* species was mainly associated with elevation, while the abundance of *A*. *firma* and *G*. *salvinii* was positively associated with soil nutrients (Fig 2B).

### Morphometric and stomatal variation at the community level

The partial correlation analyzes showed significant correlations among morphometric characters (Table 1); strong positive correlations were found between blade (BL) and stipe length (SL) and between BL and trunk diameter (TD). Stomatal traits also showed significant but weaker correlations with morphometric traits. The correlations controlled for species identity showed similar patterns of correlation among traits (*t*(14) = 0.984, *p* = .341), with the exception of the SS-TD and SS-BL relationships. The analyses of variance showed that zones and vegetation types alone were not significant factors in explaining differences in stomatal and morphometric traits (Table 2), but these analyzes showed significant interspecific differences and significant species-zone and species-vegetation type interactions for most traits.

**Table 1:** Results of the partial correlation analysis between functional characters of five tree fern species in Sierra of Juarez (Mexico).

	Stomatal size	Stomatal density	Trunk diameter	Blade length	Stipe length
Stomatal size		0.134*	0.075	-0.002	-0.105*
Stomatal density	0.138*		0.285***	0.195***	0.075
Trunk diameter	0.263***	0.337***		0.492***	-0.098
Blade length	-0.091	0.207***	0.401***		0.556***
Stipe length	-0.113*	0.075	-0.128*	0.593***	

Values above the diagonal depict the partial correlation coefficients when controlled by species. Values below the diagonal depict partial correlation coefficients without controlling for species. Significance level (p-value): *0.05, **0.01, ***0.001.

**Table 2:** Analysis of variance using linear mixed-effect models (LMM) evaluating differences between species, zones and vegetation types for five functional characters of five tree fern species.

	LMM (*F* ^p^)			LMM (*F* ^p^)			LMM (*F* ^p^)		
	Species	Plot	Interaction	Zone	Species	Interaction	Vegetation	Species	Interaction
Stomatal density (mm^2^)	5.42**	1.07	3.44***	1.16	5.03	6.14***	4.3e-6	5.41	3.64*
Stomatal size (μm)	5.06**	1.25	3.56***	1.70	7.59	1.88	0.37	11.09	1.140
Blade length (cm)	5.07**	1.32	3.89***	2.97	1.50	18.45***	9.28	0.57	26.54***
Stipe length (cm)	5.95**	0.69	4.21***	0.36	2.55	16.77***	2.45	1.93	11.21***
Trunk diameter (cm)	0.62	4.62*	1.08	1.31	2.11	11.42***	2.84	0.92	15.60***

*F* = F-statistic; p = p-value; Significance level (p-value): *0.05, **0.01, ***0.001.

**Fig 3:**
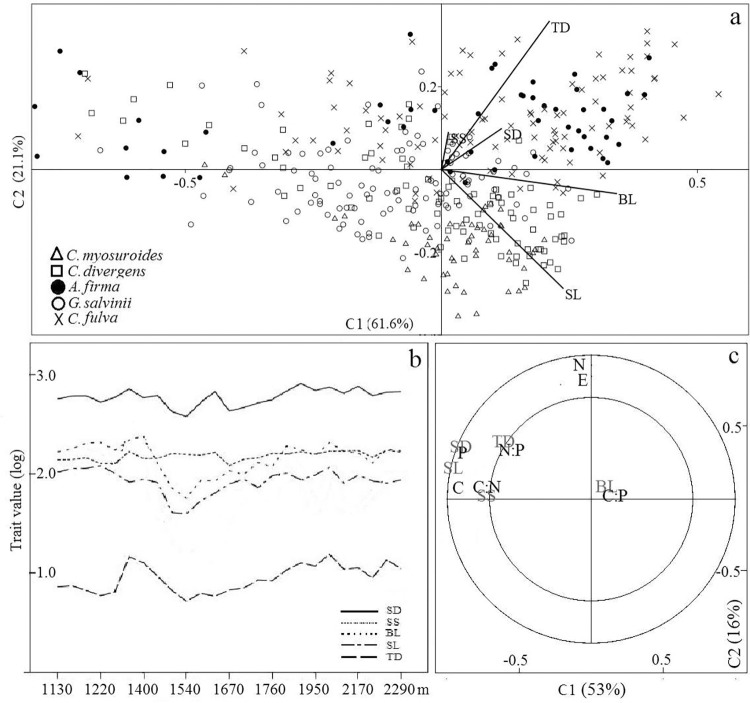
Patterns of variation of morphometric and stomatal characters of five tree ferns species along elevation and soil nutrient gradients in Sierra of Juarez (Mexico). a) PCA for five functional traits. Symbols indicate the species identity. b) Mean values over the total elevation range. c) Partial least squares regression (PLSR) showing trait—environment (elevation and soil nutrients) associations. Elevation was considered as a proxy of temperature. Explained variance is shown for each component. BL. Blade length. SL. Stipe length. TD. Trunk diameter. SD. Stomatal density. SS. Stomatal size. E. Elevation. P. Phosphorus. C. Carbon. N. Nitrogen.

The PCA resulted in a first component that explained 61.6% of variation in traits and was strongly loaded by blade length; as second component explained 21.1% of variation and was strongly loaded by trunk diameter and stipe length (Fig 3A). The analysis showed that variation along the first component was not associated with species identity and most species showed large and small leaves. The second component broadly separated *C*. *fulva* and *A*. *firma* (with wide trunks) from the rest of the species, especially from individuals of *C*. *myosuroides* that inhabit the transition zone at the bottom of the gradient. Difference in the degree of variation illustrated by the PCA was also evident when analyzing the mean values along the gradient (Fig 3B). Morphometric traits showed a tendency to decrease towards the center of the gradient, especially between the cloud forest and the transition zone; mean stomatal density showed similar pattern of variation along the gradient. According to the results of the PLSR analysis, at the community level the variation in soil nitrogen was associated with elevation, whereas morphometric traits (BL and TD) were associated with (or were predicted by) soil nutrients ratios and stomatal density was associated with soil P (Fig 3C).

### Morphometric and stomatal variation at the species level

**Fig 4:**
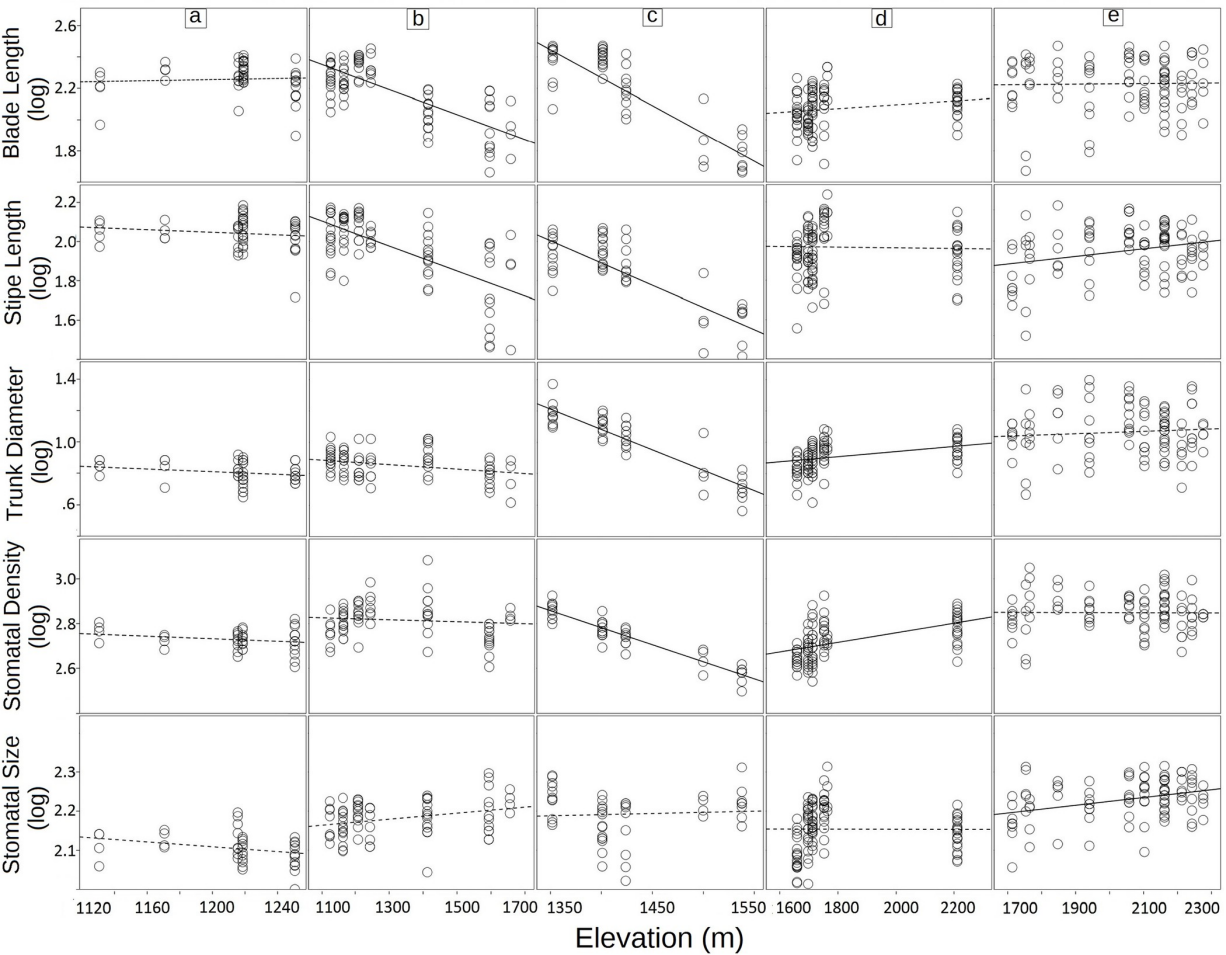
Linear Mixed-effect Models (LMM) for three morphometric and two stomatal characters along elevation ranges of five tree ferns species in Sierra of Juarez, Mexico. Solid lines indicate significant regression (trait—elevation linear relationships). a) *Cyathea myosuroides*; b) *C*. *divergens*; c*) Alsophila firma*; d) *Gymnosphaera salvinii*; e) *C*. *fulva*.

**Fig 5:**
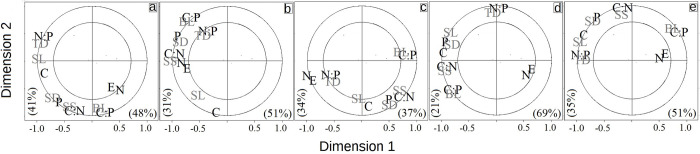
Partial Least Square Regression (PLSR) for three morphometric and two stomatal characters along elevation ranges of five tree ferns species in Sierra of Juarez, Mexico. PLSR graphs show trait—environment (elevation and soil nutrients) relationships. a) *Cyathea myosuroides*; b) *C*. *divergens*; c*) Alsophila firma*; d) *Gymnosphaera salvinii*; e) *C*. *fulva*. BL. Blade length. SL. Stipe length. TD. Trunk diameter at breast height. SD. Stomatal density. SS. Stomatal Size. E. Elevation. P. Phosphorus. C. Carbon. N. Nitrogen.

In general, the associations between traits and soil nutrients were consistent across species, but some interspecific differences were observed in the direction of these associations, as well as in the association between nutrients and elevation (Figs 4 and 5; S2 Table). The clearest trends were observed in *A*. *firma* and *C*. *divergens* that inhabit both the cloud forest and the transition zone. However, although these species share patterns for stipe length and blade length, trends for stomatal density and trunk diameter showed interesting differences, as these traits significantly decreased towards cloud forest in *A*. *firma*, while no significant trends were observed in *C*. *divergens*. For its part, *G*. *salvinii*, whose distribution is adjacent to that of *A*. *firma*, but restricted to cloud forest, showed opposite patterns for stomatal density and trunk diameter. For *C*. *fulva*, that inhabits the upper edge of the cloud forest, positive associations with elevation were revealed for stipe length and stomatal size, which were not observed in any other species. *Cyathea myosuroides* did not show significant trends.

### Contribution of intra and inter-specific variation to community response

**Fig 6:**
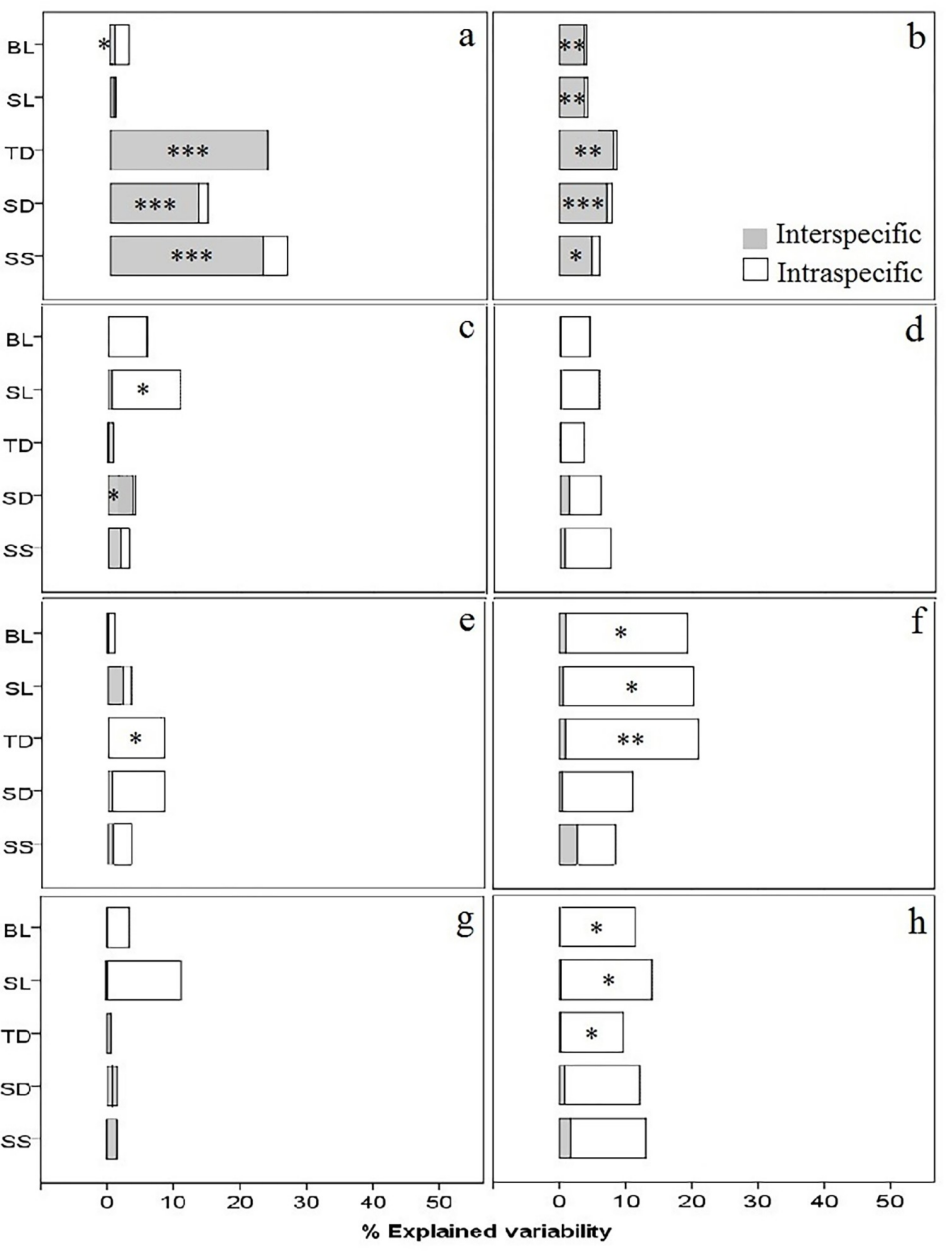
Contribution of intra- and interspecific variability of abundance-weighted and unweighted trait values of three morphometric and two stomatal traits along elevation and soil nutrient gradients in a fern community in Sierra of Juarez (Mexico). The contribution is evaluated through the percentage of explained variability respect to the total variability, which reflects the community response to: a-b. Elevation. c-d. soil C. e-f. soil P. g-h. soil N. Significance for each variability component: Interspecific, intraspecific and specific is indicated per trait. BL. Blade length. SL. Stipe length. TD. Trunk diameter. SD. Stomatal density. SS. Stomatal size. *p*-value = <0.05*, < 0.01**, <0.001***.

The contribution of inter- and intraspecific variability of morphometric and stomatal traits to the response of the tree fern community to elevation and soil gradients is shown in Fig 6 (see S3 and S4 Tables). For both abundance-weighted and unweighted trait values, we found a low contribution of intraspecific variability, but a significant contribution of interspecific variability in stomatal traits and trunk diameter to the community responses to the elevation gradient (Fig 6A and 6B). In contrast, the results showed that, for abundance weighted values, intraspecific variability in morphometric and stomatal traits had a higher relative contribution to community response to soil nutrient gradients than interspecific variability (Fig 6C–6H). For unweighted trait values, the contribution of intraspecific variability to community response to soil gradients decreases substantially for morphometric traits.

## Discussion

Here we found evidence that the distribution and abundance of tree ferns are partially determined by environmental variables and that community composition is driven by the joint effects of elevation and soil nutrients. These effects of environmental variables are reflected in a steep species turnover of tree ferns, which emerged as a main response to elevation. However, we found that variation in morphometric and stomatal traits was not explained by different vegetation types, but instead was strongly influenced by species identity and species altitudinal range. Intra-specific trait variability was relevant under contrasting environmental conditions, as the most pronounced morphometric/stomatal observed within species coincide with steep changes in relative humidity, soil nutrients, and forest structure.

The marked species turnover observed along the elevation gradient supports the strong environmental determinism of ferns in montane forest reported by previous studies [40,58–60], although recent studies attach more importance to other factors, including geographical constraints and habitat availability [61]. Although it was impossible to test the consistency of this pattern at different locations, our results seem to reflect a common pattern among tree fern communities, which show a marked elevation turnover between closely related species and even within species [32–34]. In our study, *C*. *fulva* and *C*. *divergens*, the phylogenetically closest species, illustrate these patterns of elevation-driven turnover; these two species occupy separate, but adjacent, elevation ranges. Our results also support the observations of a tendency for ferns to occur in single-species stands [Page, 1979, as cited in 28], as half of the studied sites were dominated by a single species. Evidence suggests that environmental determinism in ferns appears to be strongly influenced by topographic, climatic, and soil conditions [29,58,62]. In addition to high humidity conditions that seem to govern the distribution of tree ferns across montane regions [27,63,64], light incidence appears to be a determining factor to explain the distributions of tree ferns [29,37], especially during the first phase of the life cycle [28,38,65–67]. For instance, our results support the shade tolerance observed of *A*. *firma*, that showed a restricted distribution biased towards the cloud forest zone; in contrast, *C*. *divergens* tolerates more open sites [28,38] and showed a distribution across the entire transition zone and a considerable proportion of the cloud forest. Our results support evidence that *C*. *divergens* and *A*. *firma* are ecological indicators of environmental quality at the edge of the cloud forest [68].

Our findings revealed a possible influence of soil nutrients on the abundance and turnover of species. These is evident in at least two species of tree ferns: *A*. *firma* and *G*. *salvinii*. The elevation ranges of these two species coincide with zones of greater variability in soil nutrients; the distribution of *A*. *firma* seems to be limited to areas with high concentration of phosphorous, whereas the areas inhabited by *G*. *salvinii* have high nitrogen and carbon concentration with relatively low phosphorus. Our results reinforce evidence of the importance of soil nutrients to understanding community structure in ferns [29,58,65]. However, in our study the influence of soil nutrients was not consistent across species, since the abundance of *Cyathea* species was not associated with soil nutrients. In line with these results, a higher contribution of intra-specific variability, relative to inter-specific (turnover species) variability, to community response to soil nutrients was observed using abundance-weighted trait values. This suggest that community response to soil nitrogen and phosphorus is strongly influenced by the abundance of *A*. *firma* and *C*. *divergens* rather than by species turnover.

Inter-specific variability showed a higher contribution to morphological and stomatal variation in tree fern along elevation compared to the contribution of intra-specific variability. These results and the marked species turnover along the elevation gradient, support the hypothesis that fern communities adapt to changing temperature and humidity levels primarily through shifts in the community composition and only to a lesser degree through intraspecific plasticity [18]. However, the greater contribution of intra-specific variability for morphometric traits (BL and SL) than for stomatal traits (SD, SS) probably reflects a more limited anatomical response of species to environmental variation. Accordingly, at the community level, total trait variation was dominated by morphometric variation, which is not surprising given that these characters in ferns frequently exhibit a high degree of plasticity in response to elevation temperature, humidity and light gradients [18,67,69,70]. At the species level, morphometric trends appear to be the result of a zone-species combination rather than an elevational trend, which could greatly explain variation in blade length [18].

### Fern physiology and morphology

Variation in morphometric traits appear to be strongly influenced by allometric relationships. In the five tree fern species studied, blade length was correlated with stipe length and trunk diameter, which agrees with previous observations for the tree fern *C*. *caracasana* in the Andes [41], a species closely related to *C*. *divergens* [71]. These observations suggest a consistent allometric relationship between leaf and trunk size in tree ferns. Interestingly, trunk diameter and blade length exhibited significant correlations with stomatal traits, particularly with stomatal density.

Evidence suggests that the adaptation of fern species to different habitats may be explained by a combination of leaf hydraulic vulnerability and hydraulic adjustments of ferns fronds, both with consequences on leaf hydraulic conductance (K_leaf_) [72,73]. Among hydraulic adjustments, variation in vein density (D_vein_) has been strongly associated with K_leaf_ (via a negative correlation with hydraulic resistance of leaf), as well as with stomatal conductance, photosynthetic capacity, and stomatal density [74–76]. Zhang et al. [35] found a significant association between D_vein_ and stomatal density, even after phylogenetic correction. In turn, D_vein_ can also limit the diameter growth rates of tropical trees through constrains to the xylem water transport capacity [77]. These associations offer a way to explain the observed correlations between trunk diameter and stomatal density; adjustments to stomatal density in response to environmental changes may require (or be controlled by) adjustments to other phenotypic traits (in this case size) to keep the supply of photosynthetic resources and avoid water stress.

Similar adjustments in trunk diameter in response to variation in stomatal size could also be supported by a relationship between D_vein_ and stomatal size [78]. In our study, only *G*. *salvinii* showed a significant correlation between stomatal size and trunk diameter (r = 0.287, p = 0.004). This species exhibited relatively small stomata (length x width) at low densities (lowest), which, however, can reach relatively high stomatal conductance (S5 Table). A relatively high stomatal conductance could be due to a higher stomatal length that might require hydraulic adjustments (e. g. diameter). However in *C*. *fulva*, with similar stomatal length, the correlation between trunk diameter and stomatal size is very low (r = 0.015, p = 0.890), which suggest that this relationship may depend on the stomatal shape (strongly elongated in *G*. *salvinii*; S5 Table) and/or the environmental context. It is known that variation in stomatal shape can affect stomatal conductance and resistance to diffusion [79,80, see other references in 81]. On the other hand, the fact that *A*. *firma* (r = -0.214, p = 0.178) and *C*. *divergens* (r = -0.120, p = 0.321) showed an opposite correlation between these traits may evidence the relevance of the environmental context.

Some variation in stomatal traits at the community level was expected across the elevation range, with a steep temperature gradient and (at least) two different forests types that affect humidity and light levels. The preference for humid environments and the low control of evaporative potential in ferns in comparison with angiosperms has been well documented [36,82,83]. Gas exchange efficiency during photosynthesis is strongly determined by the interdependence between stomatal density and size, since both characters define the maximum diffusive conductance *g*_*smax*_ [84] that determines the photosynthesis process [85], although a limited ability to control stomatal opening and closing is also relevant [36]. The lower variation in stomatal size (20%) and density (15%) compared to morphometric traits (20–50%) appears to reflect higher levels of genetic determination [18,35,86]. Our results support these observations, showing that stomatal size is mostly determined by inter-specific differences (Table 2) and that variation in stomatal traits associated with environmental changes appears to be almost limited to adjustments in stomatal density. In addition, a low correlation between stomatal size and density, also observed in temperate ferns [87], suggested that these two traits are not controlled by the same mechanisms in ferns. However, this result was influenced by how we estimated stomatal size, as stomatal density was correlated with stomatal length (r = -0.261, p < 0.001) and stomatal width (r = 0.358, p < 0.001), indicating that the degree of interdependence of these stomatal traits may vary among fern species.

Contrasting environments in terms of temperature and humidity at the low edge of the cloud forest appear to have a large effect on morphometric traits. A warmer, less humid environment, and more open forest structure towards the transition zone supports plants with larger leaves (blade and stipe), while more humid and colder environment support plants with smaller leaves; this is reflected in the consistent pattern observed for *A*. *firma* and *C*. *divergens*. Accordingly, Bernabe et al. [28] found evidence of larger leaf sizes and higher growth rates at forest edges in *A*. *firma*, *Lophosoria quadripinnata*, and *Sphaeropteris horrida*. Likewise, Arens and Baracaldo [41] observed the same patterns in *C*. *caracasana* across a successional mosaic in the tropical Andes. Furthermore, our results reflect a differential effect of relative humidity on species morphology. Species restricted to the cloud forest zone, where relatively humidity is more constant, showed little to no trends in blade length compared to species that inhabit zones where relative humidity is more heterogeneous and with more pronounced changes (at the edge of the cloud forest zone). Riaño and Briones [39] observed that tree ferns within closed and more humid habitats had smaller fronds without substantial changes in size with varying levels of humidity; on the contrary, in open sites, the presence of larger fronds was influenced by humidity levels, suggesting that a combination of light availability and humidity drives variation in frond size in tree ferns. Although we could not explore the effect of light incidence, the close agreement between morphometric and stomatal trait variation at the low edge of the cloud forest zone suggests a strong influence of relative humidity and light. In ferns, shade-tolerant plants have been proposed to display low phenotypic plasticity in response to changes in light availability compared to sun-tolerant plants [see references in 88]. However, in our study, *A*. *firma*, a shade-tolerant species, showed great variation (similar to that of *C*. *divergens*, a sun-tolerant species) in both morphometric and stomatal traits among different types of vegetation with a clear contrast in light environments. Light intensity has been strongly associated with the variation of stomatal density, stomatal conductance and photosynthesis capacity in ferns [36,39,89,90]. Although it is an inconsistent pattern among tree ferns [91,92], light intensity has also been associated with higher stomatal density in *A*. *firma* and *C*. *divergens* [38]. This could explain the increase in stomatal density towards more open zones with increased light incidence in *G*. *salvinii* and *A*. *firma*. In addition, light incidence is associated with variability in blade and stipe length, in which the development of larger fronds is a strategy to compete for light resources [39,41]. However, the positive relationship between light incidence and relative growth rates is not consistent among studies [93,94].

The more pronounced pattern for stomatal density appears to reflect contrasting humidity environments at the lower edge of the cloud forest zone. Similar to the morphometric traits, stomatal density in *A*. *firma* decreases from the transition zone to the lower zone of the cloud forest. This may suggest the same regulatory mechanism for both types of traits. This mechanism could be supported, again, by the role of D_vein_. A negative association between D_vein_ and humidity conditions has been found in both angiosperms and ferns [see references in 18]. Our study did not estimate D_vein_, however, lower values of SD (strongly correlated with D_vein_ in ferns) and TD (potentially limited by D_vein_), coincide with the most humid area within the elevation range of *A*. *firma*, while a contrasting pattern arises towards the transition zone. Theoretically, high D_vein_ in the transition zone could promote higher K_leaf_, stomatal density, stomatal conductance, and higher rates of gas exchange per leaf area [74–76]. In the cloud forest, in shaded sites with a high water supply, low D_vein_ could promote “a reduced construction cost, and also correspond to a lower vein projected area and volume, and thus to less displacement or shading of photosynthetic mesophyll” [Sporck and Sack cited in 76]. Based on this, a combination between adjustments in the hydraulic system and a great capacity to compete for light resources [28,37,39] could determine the distribution of *A*. *firma*. However, not all the species are equally sensitive to changes in relative humidity [95] and in some species D_vein_ does not correlate with K_leaf_ [see references in 76]. This might explain the dissimilar patterns of variation in stomatal density and trunk diameter displayed by *C*. *divergens* and *A*. *firma* in the same area and could reflect a phylogenetic component. But, because *C myosuroides* and *C*. *fulva* were not distributed in contrasting humidity environments along the studied range, the consistency of this pattern in *Cyathea* species could not be verified. *Cyathea myosuroides* did not show significant trends for any trait probably because only the upper part of its distribution was sampled (1,100–1,250 m). Interestingly, *C*. *myosuroides* inhabits warmer and less humid areas than any other species in our study and showed the lowest combined values of stomatal size and density (S5 Table), which theoretically lead to lower stomatal conductance [84]. Evidence suggests that low stomatal conductance may represent and advantage in more arid conditions [96].

The patterns of morphometric and stomatal variation reported here allowed a partial understanding of how the phenotypic response to environmental change may be associated with community composition and structure by determining both the factors driving this variation and the adaptive potential of species in a context of interspecific competition and ecological differentiation. Environment stability in terms of light and humidity availability has a significant effect on trait-environment relationship trends at the intraspecific level and on species distribution ranges at the interspecific level. The fact that only *A*. *firma* and *C*. *divergens* inhabit both the low edge of the cloud forest and the transition zone (*A*. *firma* only a small interval) seems associated with the ability to make significant adjustments in combined morphometric and stomatal traits to maintain adequate gas exchange efficiency and photosynthetic capacity in contrasting environments. Previous evidence suggested that the performance of tree fern species is associated with their position within the forest, such that some species (e. g. *C*. *divergens*) show high growth rates (frond length) at the forest edge, while other species perform best at the interior of the forest [28]. Interestingly, in our study, the significant trends in morphometric variation in species distributed within the cloud forest seem to respond to other environmental factors (a decrease in temperature and a theoretical increase in light incidence towards the top of the gradient). Derived from the above discussion, it is clear that local environmental selection promotes different phenotypic adjustments along the elevation gradient, establishing a relationship between elevation and the type and level of variation that allows for the formulation of hypotheses about how species might respond to different environmental scenarios (e. g. due to climate change), and how the community structure could be affected. For example, although ferns have a reduced ability to control stomatal opening and closing compared to angiosperms, distribution ranges of *C*. *fulva*, *C*. *divergens* and *C*. *myosuroides*, from top to bottom of elevation gradient, may be associated with stomatal size, where large stomata (e. g. *C*. *fulva*) have been associated with low responsiveness in rapidly changing conditions and small stomata (e. g. *C*. *myosuroides*) have been associated with conditions of higher rates of gas exchange [97].

### Correlations with soil nutrients

Contribution of phenotypic variability to community response to soil nutrients appears to be trait- and nutrient-specific. While blade and stipe length variability contributed mainly to the response to soil carbon and nitrogen, the variability of trunk diameter and stomatal density contributed mainly to the response to soil phosphorous (Fig 6). The community response to soil nutrient variability is supported by the association between blade length and trunk diameter with C:P and N:P ratios, respectively. The association between soil nutrients ratios and morphometric traits is not surprising, since plant growth largely depends on nutrient ratios, especially N:P that is a key element for the photosynthesis process and modulation of soil carbon storage [50], promoting plant growth by affecting soil properties [98]. Furthermore, soil fertility (C:N:P ratios) has been shown to have a significant effect on the functional diversity of tropical ferns [99].

Our results support observations that stem diameter increases in response to soil phosphorus [100,101, see other references in 102]. This is probably linked to an increase in leaf area and photosynthetic capacity [101,103,104], which in turn can be achieved through adjustments to stomatal density [105]. In our study, an increase in elevation coincides with a decrease in soil nutrients, especially soil phosphorus at the lower edge of the cloud forest zone. Although concentrations of soil nutrients in this zone were higher compared to other areas of the gradient, plant growth in *C*. *divergens* and *A*. *firma* could be strongly determined by phosphorus and nitrogen concentrations, as observed in other tree ferns (*Cibotium glaucum*) [106].

## Conclusions and perspectives

Our results indicate that the distribution and abundance of tree ferns across elevation are partly determined by variation in relative humidity and soil nutrients. Species turnover and the corresponding change in morphometric and stomatal traits appears to be the main community response to the elevation gradient, whereas intra-specific variation appears to be linked to soil nutrients gradients. Morphometric and stomatal traits showed differences in the extent of variation and intraspecific variability, which was high across contrasting environmental conditions; the clearest trends were observed at the lower edge of the cloud forest zone that coincides with the most pronounced changes in relative humidity and soil nutrients. These intra-specific responses to environmental gradients suggest a great adaptive potential of the tree fern community to changing environmental conditions. Furthermore, similar trends in morphometric traits among species distributed within the same elevation range, suggest consistent responses to environmental changes, but inconsistencies in traits coupled with hydraulic properties may indicate different adaptive strategies, probably through different stomatal and hydraulic adjustments.

Taken together, our findings provide evidence of a likely diversity of adaptive strategies among tree fern species in response to different levels of environmental change. These responses to abiotic factors have consequences on species diversity and richness in an area of high conservation priority of tree ferns in Mexico. Our results reveal a strong relationship between elevation and the type (trait) and level of variation, which supports the need to protect the entire elevation range studied and maintain this elevational strip as a bastion of functional and species diversity in tree ferns, especially considering that the lower part of the gradient (represented by the tropical rain forest), which was not included in this study, has been strongly impacted by anthropic activities. Fortunately, this situation may provide an opportunity to assess the impact of high habitat disturbance on morphological variation patterns, but also on other functional traits in this same gradient. On the one hand, morphometric and stomatal variation, as well as the environmental preferences of the species, can be closely linked to biological cycles highly influenced by climate variation, so it is desirable to include phenological traits in future studies to determine whether intra- and interspecific variation of these traits can explain patterns of differentiation between species and populations. On the other hand, considering that climate change has been associated with the likely elevational migration of tree fern species [107], studies that address the causes of the coexistence of different species of tree ferns in a given site or the effect of interspecific competition will be of great relevance. This information would be essential to propose hypotheses about changes in the structure of communities due to environmental (climate) change phenomenon.

Our exploratory analysis did not show significant relationships between environmental factors and the presence of two or more species in the sampling sites, but studies focused on this issue should be developed. In addition, an important component of the adaptive potential in response to environmental change is the genetic potential for local adaptation. The study of genetic variation will not only allows the development of hypotheses about the adaptive capacity of populations and species at different sites along the gradient, but will also provide information about the process driving genetic differentiation and isolation between populations associated with environmental change.

The ability of tree fern species to survive or establish in a given area does not depend initially on the ability to make adjustments to leaf size and stomatal density or other sporophyte characteristics, but on the success of germination and survival of gametophytes. This indicates the need to explore early life-stage variation and performance across environmental gradients with the aim of carrying out a comprehensive analysis. In particular, it is essential to investigate and determine the physiological tolerance ranges of tree fern gametophytes compared to sporophytes. Ultimately, whatever the life stage studied, the role of soil nutrients should not be ignored, because these appear to be determinants of tree fern species abundance and sustain phenotypic variation among and within populations.

## Supporting information

S1 Fig *In situ* photographs showing the consequences of disturbance by strong winds in Sierra of Juarez (Mexico). a) Image showing one of the many large gaps caused by the weather event. b) Image showing the exposed roots of a very large tree to reflect the intensity of the weather event.
(PDF)

S2 Fig Ratios of three main soil nutrients along an elevation gradient in Sierra of Juarez (Mexico). (PDF)

S1 Table Results of the analysis of variance to assess differences in soil nutrients between zones and between types of vegetation of an elevation gradient located in Sierra of Juarez (Mexico). df. Degrees of freedom. MS. Mean squares. F. *F*-Statistic. p. Significance.
(PDF)

S2 Table Results of regression analysis at the species level using linear mixed-effects models. *F*-statistic and the *p*-value are shown only for the simple linear model. Only significant regressions are shown. BL. Blade length. SL. Stipe length. TD. Trunk diameter. SD. Stomatal density. SS. Stomatal size. E. Elevation. C. Carbon. N. Nitrogen. P. Phosphorus. R^2^_._ Adjusted determination coefficient. F. *F*-Statistic. *p*. Significance. *SD. Standard deviation of random effects. Lower-95 and Upper-95. Confidence intervals for LMM. Elevation (*β*). Parameter coefficient.
(PDF)

S3 Table Results of regression analyzes applied to three groups of average abundance-unweighted trait values of three morphometric and two stomatal characters for five tree fern species distributed in Sierra of Juarez (Mexico). Trait ‘specific’ values were calculated by averaging the species traits values in that plot. Trait ’fixed’ values were obtained averaging the species traits values obtained by averaging the species values of all plots in which a species occurs. The traits intraspecific values are the result of subtracting the fixed trait values from the specific trait values. Covariance is calculated according to equation 1.1, as SS cov = SS specific- (SS fixed + SS intraspecific). BL. Blade length. SL. Stipe length. TD. Trunk diameter. SD. Stomatal density. SS. Stomatal size. Elev. Elevation. SS. Sum of squares. DF. Degrees of freedom. MS. Mean squares. F. *F*-Statistic. p. Significance. cov. Covariance.
(PDF)

S4 Table Results of regression analyzes applied to three groups of average abundance-weighted trait values of three morphological and two stomatal characters for five tree fern species distributed in Sierra of Juarez (Mexico). Trait ‘specific’ values were calculated by averaging the species traits values in that plot. Trait ’fixed’ values were obtained averaging the species traits values obtained by averaging the species values of all plots in which a species occurs. The traits intraspecific values are the result of subtracting the fixed trait values from the specific trait values. Covariance is calculated according to equation 1.1, as SS cov = SS specific- (SS fixed + SS intraspecific). BL. Blade length. SL. Stipe length. TD. Trunk diameter. SD. Stomatal density. SS. Stomatal size. Elev. Elevation. SS. Sum of squares. DF. Degrees of freedom. MS. Mean squares. F. *F*-Statistic. p. Significance. cov. Covariance.
(PDF)

S5 Table Mean value and standard deviation of three morphometric and two stomatal characters of five tree fern species distributed along an elevation gradient in Sierra of Juarez, Mexico. Mean ± SD (cv)^post hoc difference^; N = sample size; W_s_ = Width of stomata; L_s_ = Length of stomata. BL. Blade length. SL. Stipe length. TD. Trunk diameter. SD. Stomatal density. SS. Stomatal size. * Theoretical estimation based on Franks & Beerling (2009) [84].
(PDF)
