# Supplementary figures and images for: Distribution and morphological variation of tree ferns (Cyatheaceae) along an elevation gradient
Source: PLoS One. 2023 Sep 27;18(9):e0291945. doi: 10.1371/journal.pone.0291945 (PMC10530041; doi:10.1371/journal.pone.0291945)

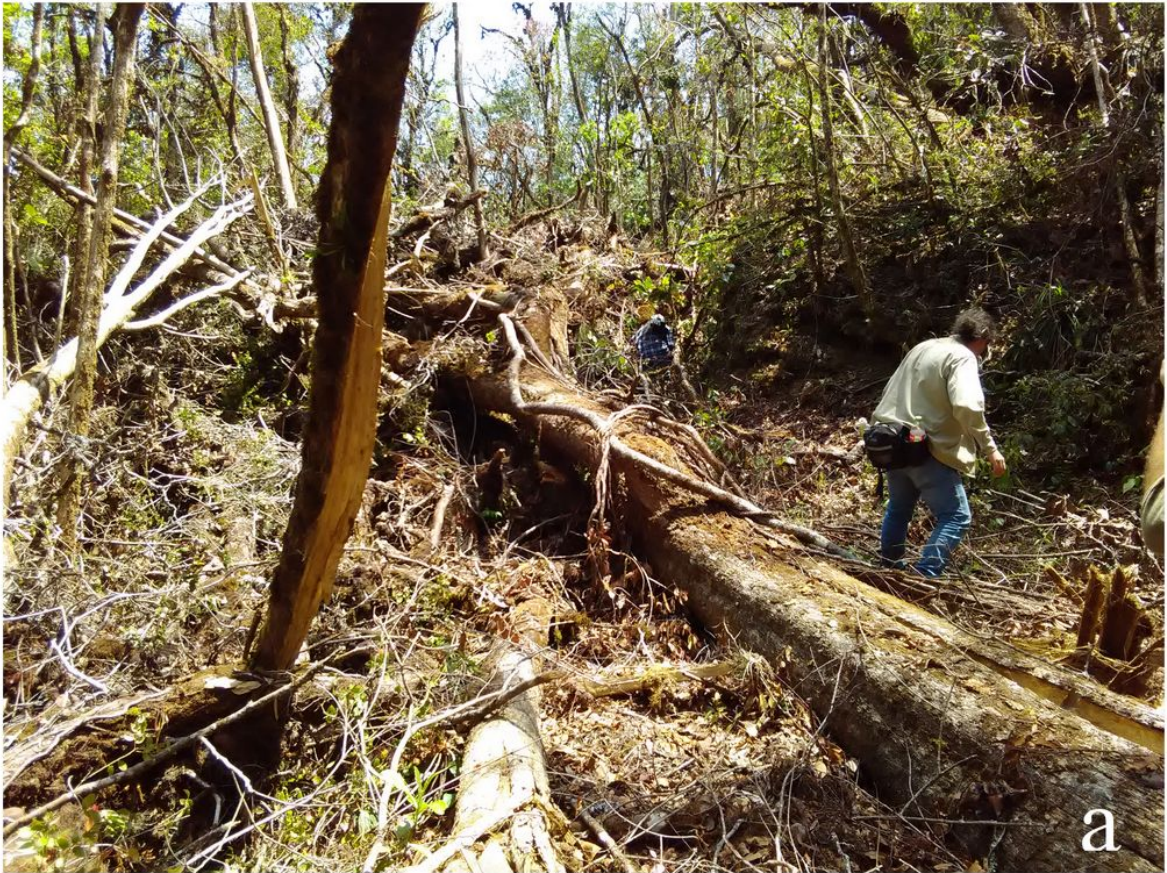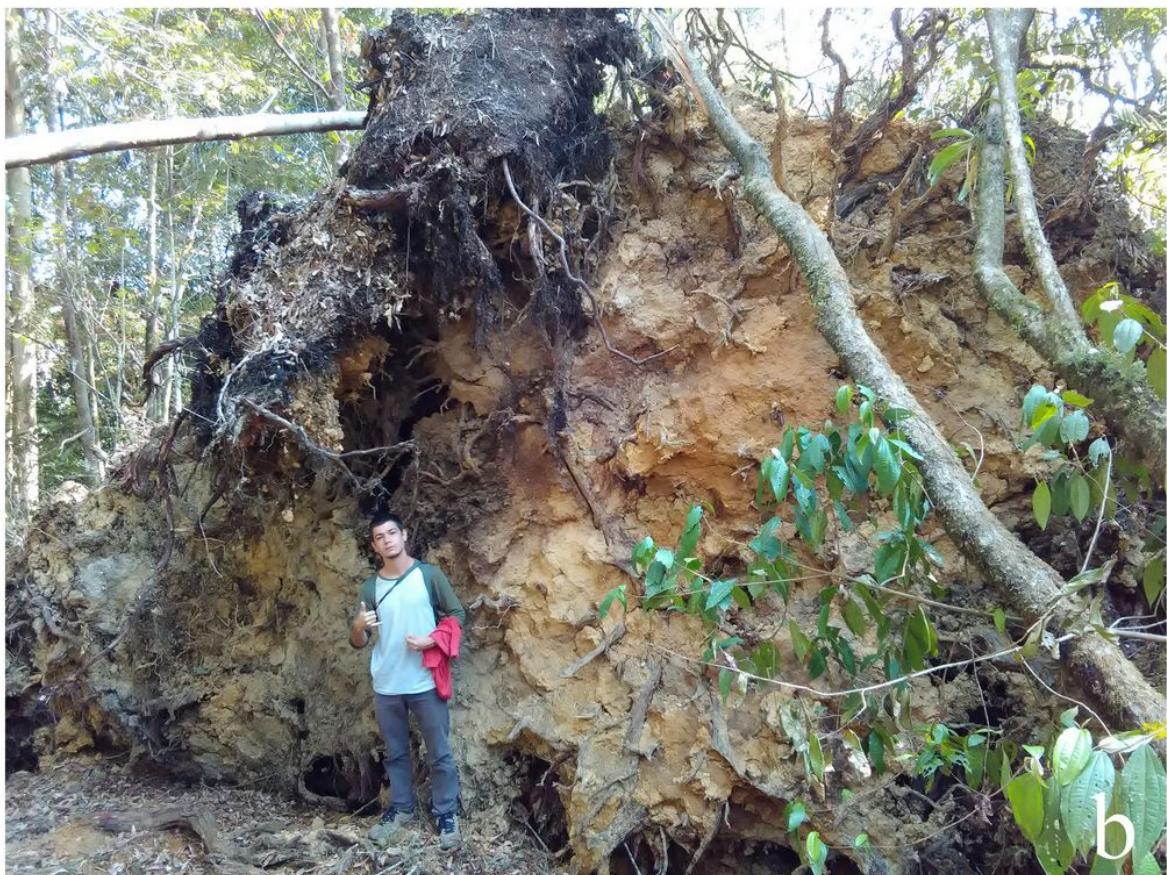

Supplement: S1 Fig — a) Image showing one of the many large gaps caused by the weather event. b) Image showing the exposed roots of a very large tree to reflect the intensity of the weather event. (PDF) [file pone.0291945.s001.pdf]

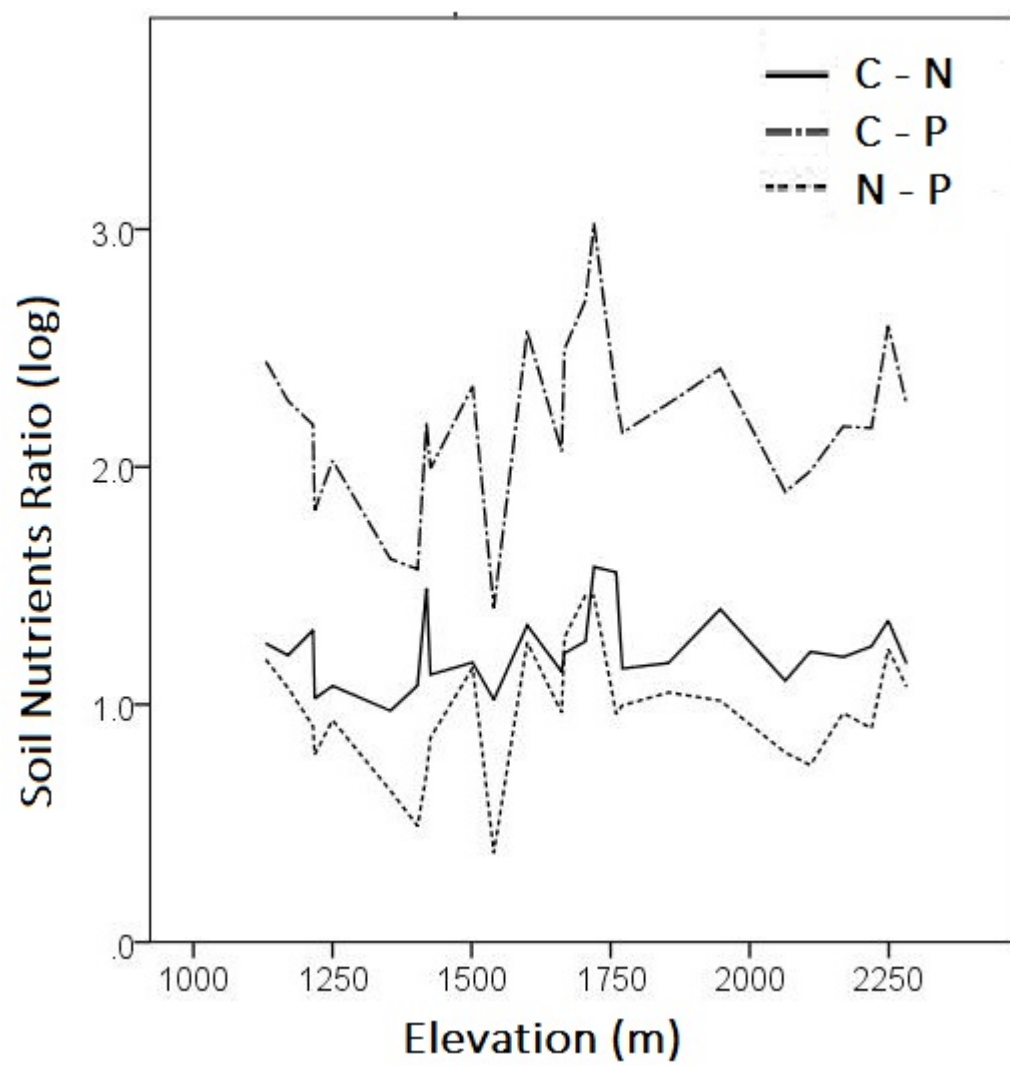

Supplement: S2 Fig — (PDF) [file pone.0291945.s002.pdf]
