# Supplementary material for: Distribution and morphological variation of tree ferns (Cyatheaceae) along an elevation gradient
Source: PLoS One. 2023 Sep 27;18(9):e0291945. doi: 10.1371/journal.pone.0291945 (PMC10530041; doi:10.1371/journal.pone.0291945)
Supplement: S1 Table — df. Degrees of freedom. MS. Mean squares. F. F-Statistic. p. Significance. (PDF) [file pone.0291945.s003.pdf]

**Group**	Response Variable	df	MS	F	p
Zone	Ct	2	.179	1.700	.205
	Nt	2	.099	1.530	.238
	Pt	2	.020	.350	.709
	C:N	2	.027	1.130	.340
	C:P	2	.263	2.257	.127
	N:P	2	.156	2.510	.103
Vegetation	Ct	1	.243	2.296	.143
	Nt	1	.095	1.428	.244
	Pt	1	.040	.716	.406
	C:N	1	.039	1.627	.214
	C:P	1	.429	3.703	.066
	N:P	1	.230	3.658	.068
