# Supplementary material for: Distribution and morphological variation of tree ferns (Cyatheaceae) along an elevation gradient
Source: PLoS One. 2023 Sep 27;18(9):e0291945. doi: 10.1371/journal.pone.0291945 (PMC10530041; doi:10.1371/journal.pone.0291945)
Supplement: S2 Table — F-statistic and the p-value are shown only for the simple linear model. Only significant regressions are shown. BL. Blade length. SL. Stipe length. TD. Trunk diameter. SD. Stomatal density. SS. Stomatal size. E. Elevation. C. Carbon. N. Nitrogen. P. Phosphorus. R2. Adjusted determination coefficient. F. F-Statistic. p. Significance. *SD. Standard deviation of random effects. Lower-95 and Upper-95. Confidence intervals for LMM. Elevation (β). Parameter coefficient. (PDF) [file pone.0291945.s004.pdf]

Trait	Species	R^2	Elevation (β) or *F*	Lower-95 or *p*	Upper-95	SD * (random effect)
BL	*C. myosuroides*	-	-	-	--	-
	C. divergens	0.546	-0.0007952	-0.00110096	-0.00048944	0.07014
	A. firma	0.719	-0.0035556	-0.004868016	-0.002243184	0.09278
	G. salvinii	-	-	-	--	-
	C. fulva	-	-	-	--	-
SL	*C. myosuroides*	-	-	-	--	-
	C. divergens	0.429	54.44	2.542e-10	-	-
	A. firma	0.596	-0.0022763	-0.003246696	-0.001305904	0.06491
	G. salvinii					
	C. fulva	0.077	0.0002424	1.4648e-05	0.000470152	0.05314
TD	*C. myosuroides*	-	-	-	--	-
	C. divergens	-	-	-	-	-
	A. firma	0.827	211.9	2.2e-16	-	-
	G. salvinii	0.03027	4.027	0.04758	-	-
	C. fulva					
SD	*C. myosuroides*	-	-	-	--	-
	C. divergens	-	-	-	-	-
	A. firma	0.870	294.9	2.2e-16		
	G. salvinii	0.190	1.774e-04	3.10272e-05	0.0003237728	0.03508
	C. fulva					
SS	*C. myosuroides*	-	-	-	-	-
	C. divergens	-	-	-	-	-
	A. firma	-	-	-	-	-
	G. salvinii	-	-	-	-	-
	C. fulva	0.1034	11.61		0.0009792	
