# Supplementary material for: Distribution and morphological variation of tree ferns (Cyatheaceae) along an elevation gradient
Source: PLoS One. 2023 Sep 27;18(9):e0291945. doi: 10.1371/journal.pone.0291945 (PMC10530041; doi:10.1371/journal.pone.0291945)
Supplement: S4 Table — Trait ‘specific’ values were calculated by averaging the species traits values in that plot. Trait ’fixed’ values were obtained averaging the species traits values obtained by averaging the species values of all plots in which a species occurs. The traits intraspecific values are the result of subtracting the fixed trait values from the specific trait values. Covariance is calculated according to equation 1.1, as SS cov = SS specific- (SS fixed + SS intraspecific). BL. Blade length. SL. Stipe length. TD. Trunk diameter. SD. Stomatal density. SS. Stomatal size. Elev. Elevation. SS. Sum of squares. DF. Degrees of freedom. MS. Mean squares. F. F-Statistic. p. Significance. cov. Covariance. (PDF) [file pone.0291945.s006.pdf]

		Fixed					Specific					Intraspecific					
		SS	DF	MS	F	p	SS	DF	MS	F	p	SS	DF	MS	F	p	cov
BL	Elev	0.03	1	0.03	1.01	0.33	0.56	1	0.56	1.63	0.21	0.34	1	0.34	1.18	0.29	0.19
	Ct	0	1	0	0.12	0.73	0.4	1	0.4	1.14	0.3	0.47	1	0.47	1.66	0.21	-0.08
	Pt	0.09	1	0.09	3.94	0.06	2.44	1	2.44	9.26	0.01	1.58	1	1.58	6.65	0.02	0.77
	Nt	0	1	0	0.09	0.76	1.2	1	1.2	3.81	0.06	1.09	1	1.09	4.22	0.05	0.11
	error	0.48	21				4.92	21				4.75	21				-0.31
	total	0.66	25				8.75	25				7.27	25				0.82
SL	Elev	0.03	1	0.03	3.19	0.09	0.44	1	0.44	1.73	0.2	0.24	1	0.24	1.01	0.33	0.18
	Ct	0	1	0	0.28	0.6	0.48	1	0.48	1.9	0.18	0.41	1	0.41	1.8	0.19	0.07
	Pt	0.03	1	0.03	2.71	0.11	1.77	1	1.77	8.8	0.01	1.35	1	1.35	7.22	0.01	0.39
	Nt	0.01	1	0.01	1.15	0.29	1.16	1	1.16	5.14	0.03	0.93	1	0.93	4.56	0.04	0.22
	error	0.2	21				3.71	21				3.76	21				-0.25
	total	0.28	25				6.6	25				5.85	25				0.48
TD	Elev	0.09	1	0.09	7.09	0.01	0.29	1	0.29	3.45	0.08	0.06	1	0.06	0.95	0.34	0.14
	Ct	0	1	0	0	0.96	0.1	1	0.1	1.06	0.31	0.09	1	0.09	1.57	0.22	0
	Pt	0.03	1	0.03	2.12	0.16	0.68	1	0.68	10.04	0	0.42	1	0.42	9.15	0.01	0.23
	Nt	0	1	0	0.26	0.62	0.28	1	0.28	3.37	0.08	0.22	1	0.22	4.06	0.06	0.06
	error	0.24	21				1.16	21				0.95	21				-0.03
	total	0.39	25				2.31	25				1.52	25				0.4
SD	Elev	0.03	1	0.03	9.12	0.01	0.24	1	0.24	3.52	0.07	0.11	1	0.11	1.57	0.22	0.11
	Ct	0.01	1	0.01	1.61	0.22	0.21	1	0.21	3	0.1	0.14	1	0.14	2.17	0.15	0.06
	Pt	0.02	1	0.02	7.59	0.01	0.29	1	0.29	4.33	0.05	0.15	1	0.15	2.21	0.15	0.12
	Nt	0.01	1	0.01	2.11	0.16	0.34	1	0.34	5.31	0.03	0.25	1	0.25	3.96	0.06	0.09
	error	0.05	21				1.21	21				1.33	21				-0.17
	total	0.1	25				1.9	25				1.74	25				0.06
SS	Elev	0.03	1	0.03	5.12	0.03	0.16	1	0.16	2.92	0.1	0.06	1	0.06	1.08	0.31	0.08
	Ct	0.01	1	0.01	1.33	0.26	0.18	1	0.18	3.27	0.08	0.11	1	0.11	2.2	0.15	0.06
	Pt	0.03	1	0.03	5.79	0.02	0.24	1	0.24	4.69	0.04	0.1	1	0.1	2.04	0.17	0.11
	Nt	0.01	1	0.01	2.36	0.14	0.32	1	0.32	6.68	0.02	0.21	1	0.21	4.4	0.05	0.11
	error	0.09	21				0.89	21				1.01	21				-0.21
	total	0.15	25				1.48	25				1.32	25				0.01
