# Supplementary material for: Distribution and morphological variation of tree ferns (Cyatheaceae) along an elevation gradient
Source: PLoS One. 2023 Sep 27;18(9):e0291945. doi: 10.1371/journal.pone.0291945 (PMC10530041; doi:10.1371/journal.pone.0291945)
Supplement: S5 Table — Mean ± SD (cv)post hoc difference; N = sample size; Ws = Width of stomata; Ls = Length of stomata. BL. Blade length. SL. Stipe length. TD. Trunk diameter. SD. Stomatal density. SS. Stomatal size. * Theoretical estimation based on Franks & Beerling (2009) [84]. (PDF) [file pone.0291945.s007.pdf]

Species	N	Stomatal density (mm^{-2})	g_{smax}*	Stomatal size (μm^{2})		
				W_s x L_s	L_s	W_s
Cyathea myosuroides	50	134 ± 14 (0.1)^{c}	1.96 ± 0.27	509 ± 47 (0.1)^{d}	30 ± 2 (25-34)	16.90 ± 1.08
Cyathea divergens	90	165 ± 31 (0.2)^{b}	2.41 ± 0.45	613 ± 69 (0.1)^{c}	31 ± 2 (25-39)	19.50 ± 1.43
Alsophila firma	50	141 ± 33 (0.2)^{c}	2.27 ± 0.55	628 ± 87 (0.1)^{c}	33 ± 3 (27-39)	18.77 ± 1.99
Gymnosphaera salvinii	103	132 ± 26 (0.2)^{c}	2.45 ± 0.52	579 ± 84 (0.1)^{b}	36 ± 3 (30-44)	15.92 ± 1.62
Cyathea fulva	99	180 ± 31 (0.2)^{a}	2.99 ± 0.52	697 ± 73 (0.1)^{a}	35 ± 2 (30-39)	19.82 ± 1.56
Species	N	Blade length (cm)		Stipe length (cm)		Trunk diameter (cm)
Cyathea myosuroides	50	186 ± 44 (0.2)^{a}		112 ± 19 (0.2)^{a}		6.4 ± 1 (0.1)^{d}
Cyathea divergens	90	165 ± 61 (0.4)^{a}		101 ± 32 (0.3)^{b}		7.4 ± 2 (0.2)^{c}
Alsophila firma	50	186 ± 86 (0.5)^{a}		76 ± 26 (0.3)^{c}		11.4 ± 4 (0.4)^{a}
Gymnosphaera salvinii	103	123 ± 32 (0.3)^{b}		93 ± 27 (0.3)^{b}		8.2 ± 2 (0.2)^{b}
Cyathea fulva	99	181 ± 60 (0.3)^{a}		93 ± 30 (0.3)^{b}		12.4 ± 5 (0.4)^{a}
